# The Effect of Structural Complexity, Prey Density, and “Predator-Free Space” on Prey Survivorship at Created Oyster Reef Mesocosms

**DOI:** 10.1371/journal.pone.0028339

**Published:** 2011-12-01

**Authors:** Austin T. Humphries, Megan K. La Peyre, Gary A. Decossas

**Affiliations:** 1 School of Renewable Natural Resources, Louisiana State University AgCenter, Baton Rouge, Louisiana, United States of America; 2 Department of Zoology and Entomology, Rhodes University, Grahamstown, South Africa; 3 United States Geological Survey, Louisiana Fish and Wildlife Cooperative Research Unit, School of Renewable Natural Resources, Louisiana State University AgCenter, Baton Rouge, Louisiana, United States of America; Australian Wildlife Conservancy, Australia

## Abstract

Interactions between predators and their prey are influenced by the habitat they occupy. Using created oyster (*Crassostrea virginica*) reef mesocosms, we conducted a series of laboratory experiments that created structure and manipulated complexity as well as prey density and “predator-free space” to examine the relationship between structural complexity and prey survivorship. Specifically, volume and spatial arrangement of oysters as well as prey density were manipulated, and the survivorship of prey (grass shrimp, *Palaemonetes pugio*) in the presence of a predator (wild red drum, *Sciaenops ocellatus*) was quantified. We found that the presence of structure increased prey survivorship, and that increasing complexity of this structure further increased survivorship, but only to a point. This agrees with the theory that structural complexity may influence predator-prey dynamics, but that a threshold exists with diminishing returns. These results held true even when prey density was scaled to structural complexity, or the amount of “predator-free space” was manipulated within our created reef mesocosms. The presence of structure and its complexity (oyster shell volume) were more important in facilitating prey survivorship than perceived refugia or density-dependent prey effects. A more accurate indicator of refugia might require “predator-free space” measures that also account for the available area within the structure itself (i.e., volume) and not just on the surface of a structure. Creating experiments that better mimic natural conditions and test a wider range of “predator-free space” are suggested to better understand the role of structural complexity in oyster reefs and other complex habitats.

## Introduction

Structural complexity is the physical arrangement of objects in space [1], and is a fundamental property of all ecological systems. By varying the availability and type of microhabitats available, structural complexity may influence predator-prey interactions and have significant impacts on the local faunal community (e.g., [2], [3], [4], [5], [6]). For example, habitats that are more complex often contain a greater diversity of refuges from predators [5], [7] and greater diversity (or amount) of food resources [8] thereby reducing the intensity of interaction strengths. Increased structural complexity may alter encounter rates between predators and their prey, often decreasing predation risk by interfering with predator maneuverability and/or the ability to visually detect prey [7], [9], [10]. Furthermore, by decreasing the visibility of predators or obstructing prey movement, structural complexity may negatively impact prey survivorship [11], [12], [13]. Understanding the potential effects of habitat structure and its complexity on prey survivorship is becoming increasingly important as anthropogenic and climate-induced stressors are significantly changing the physical and ecological structure of many ecosystems.

Across multiple habitat types, studies have demonstrated that increased prey survivorship occurs with increasing structural complexity, but often with diminishing returns as complexity continues to increase [14], [15], [16], [17], [18], [19], [20], [21]. In contrast to most previous experiments, two recent studies scaled predator and prey densities to the density, or amount of available habitat (submerged aquatic vegetation) using the rationale that naturally occurring complex habitats often have higher densities of animals when compared to simple habitats [22], [23]. Neither of these studies found increased structural complexity to consistently lead to greater prey survivorship [22], [23]. Field studies also differ in their conclusions with some finding greater survivorship in more complex habitats [24], [25], [26], while others have demonstrated that the effects of structural complexity may vary by the type of fish, or the type of habitat being examined [13]. Clearly, the interaction of predator-prey dynamics and structural complexity is multifaceted, and patterns may differ depending on the species of interest, habitat type examined, range of structural complexity tested, and even the definition (or method of measurement) of complexity used.

Creating standard methods for objectively describing structural complexity within and across systems has proved to be difficult [18], [27], [28], [29], [30], [31]. Complexity may include a quantitative measure of the amount or density of structure itself, as well as a qualitative measure of the heterogeneity or diversity of structures [32]. Both measures depend on the scale in which they are ecologically relevant [27]. Fractal geometry is one suggested quantitative measure of structural complexity because of its multi-scale nature and applicability across systems [33]. This method is independent of the nature of a habitat and describes an object's surface and whether it becomes more convoluted (and thus approaching a 3-dimensional object) [31]. However, the resolution at which one measures the fractal geometry may significantly influence results and therefore makes it difficult to determine scale a priori [34]. An alternative method of quantifying structural complexity may be achieved by measuring the frequency and size of interstitial spaces between units of structure along vertical and horizontal axes (Sp) along the surface of the structure [28]. This method may then be extended to include predator size (Pr) [18]. The result is a dimensionless index (Sp/Pr) that describes the perceived amount of prey refuge, or amount of interstitial space where prey are safe from predation (‘predator-free space’). In other words, Sp/Pr is intended to quantify the functional result of the structural complexity within a habitat (amount of refuge available to the organism of interest) as experienced by the organism(s), and is not simply a physical measurement. This index has been shown to be strongly related to macroinvertebrate abundance and species richness which further validates its usefulness in quantifying structural complexity [31].

In this study, we examined the relationship between prey survivorship and habitat structure and its complexity, and whether these interactions are influenced by prey density or the amount of ‘predator-free space’. For the purposes of this study, ‘structure’ refers to any object in space, whereas ‘structural complexity’ refers to the morphological characteristics within a structure itself, or the arrangement of objects in space [1]. We chose the Sp/Pr index as a measure of ‘predator-free space’ as opposed to fractals because of its generality to all structures, its ease of measurement, the lack of significant spatial scale effects, and its high correlation with species abundance and richness in a natural setting [31]. In the lab, we used oyster (*Crassostrea virginica*, hereafter ‘oyster’) shell to create mesocosm reefs with different levels of structural complexity, then quantified the survivorship of grass shrimp (*Palaemonetes pugio*) in the presence of a predator (wild red drum, *Sciaenops ocellatus*). As an ecosystem engineer, oysters and the reefs they create are hypothesized to provide many ecosystem services [35], including altering local species composition [36] or facilitating increased prey survivorship.

The goal of this study was to test the hypothesis that oyster reefs enhance prey survivorship (i.e., the presence of structure per se), and to examine how further changing reef structural complexity, prey density, and ‘predator-free space’ may impact actual prey survivorship. The first experiment (Expt 1) used a fixed number of predator and prey individuals across treatments of variable levels of structural complexity. This experiment was designed to test for the effects of structure (per se) and increasing structural complexity on prey survivorship. The second experiment (Expt 2) used the same structural treatments as the first experiment, but scaled the density of prey across treatments. This allowed testing for the effects of prey density on prey survivorship with structure present and across different structural complexities. The third experiment (Expt 3) used the same prey density treatments as the second experiment, but altered the amount of ‘predator-free space’ (by increasing interstitial space) in each structural treatment. This was designed to test for the effects of ‘predator-free space’ on prey survivorship.

## Materials and Methods

### Ethics Statement

All necessary permits were obtained for the described study. We operated under a scientific collection permit from the Louisiana Department of Wildlife and Fisheries to Dr. Megan La Peyre (S-03-2009, S-105-OYS-2010). No endangered or protected species were collected during this project. Furthermore, nekton were collected under the Institutional Animal Care and Use Committee permit 08-005 to Dr. Megan La Peyre through the Louisiana State University Institutional Animal Care and Use Committee.

### Predator and Prey Species

The red drum is a common, estuarine-dependent species that reaches its greatest abundance in the northern Gulf of Mexico (GOM) [37] and may use oyster reefs as feeding habitat [38]. It is an opportunistic feeder throughout all life stages [39] and uses mechanoreception as its primary foraging technique, with vision used secondarily [40]. The size and composition of prey consumed by red drum remain relatively constant with increasing body size [41]. When available, shrimp species (e.g., *Litopenaeus setiferus*, *Palaemonetes pugio*, *Penaeus aztecus*) often constitute the bulk of red drum diet [39].

Shrimp belonging to the genus *Palaemonetes* are among the most abundant and ecologically dominant species in coastal estuaries of the southeastern United States [42]. As potential prey items and detritivores, grass shrimp represent a vital link in the energy transfer of tidal marsh ecosystems [43]. In the presence of predators, grass shrimp select oyster-shell pyramids over seagrass and shallow water habitats as refuge [44].

### Collection and Maintenance of Experimental Species

Red drum (32.6±2.3 cm) were captured at Rockefeller State Wildlife Refuge in Grand Chenier, Louisiana, or near Cocodrie, Louisiana, using hook and line. Grass shrimp (30.9±5.9 mm) were collected along marsh edges at Caillou (Sister) Lake in Terrebonne Parish, Louisiana, Cypremort Point State Park in St. Mary Parish, Louisiana, and near Cocodrie, Louisiana, using a seine (5 x 2 m). Fish and shrimp were collected in February, 2009, for Expt 1, and in April, 2010, for Expt 2 and 3. Organisms were transported to the laboratory and held in cylindrical, recirculating fiberglass tanks (350 L) equipped with bio-filters (AST Bead Filter, Aquaculture Systems Technologies, LLC, New Orleans, Louisiana) for 2 weeks before trials were initiated. In these tanks, salinity was maintained between 13 and 17 (14.9±0.2), temperature between 22 and 28°C (26.4±0.4), ammonia less than 0.15 ppm, and oxygen concentrations between 6.1 and 9.0 mg L^−^^1^ (6.6±0.2) using multiple air stones. Fluorescent lights (40W) were placed above the holding tanks and a 12:12 h light-dark regime was maintained throughout the experiments. Individual red drum was kept in isolation in separate tanks while grass shrimp were grouped together in two holding tanks. Red drum were fed frozen penaeid shrimp, and grass shrimp were fed wet cat food.

### Experimental Mesocosms

All trials were conducted in 2 recirculating, rectangular fiberglass tanks (length x width x height: 180×90×40 cm) located side by side in a room adjacent to the holding tanks (Table 1). The bottom of each experimental tank was left bare, and water depth was maintained at 35 cm. Water quality characteristics (salinity, temperature, dissolved oxygen, ammonia concentration) were measured before and after each trial, and fluorescent lights (40 W) were placed above the experimental tanks and operated on a 12:12 h light-dark regime. A clear plexiglass cover was placed on top of each experimental tank to prevent escape by either species. All three experiments had a control treatment which consisted of a clean tank with no shell. For Expt 1 and 2, three other treatments were created by piling clean, unaggregated oyster shells in a pyramid shape (Table 1), with an average gap size in the structure of 26.3×24.7 mm. This method of reef creation was intended to mimic oyster reefs commonly found in the northern GOM where reefs have very few vertically oriented oysters due to the extensive oyster harvest activities coast-wide. For Expt 3, two other treatments were created by cementing shells together to create a structure with increased interstitial space between shells (Table 1), with an average resulting gap size of 48.4×79.4 mm. This method was used to mimic conditions of healthy reefs in the northern GOM where oysters primarily grow in a vertical orientation. A ‘high’ treatment was not created in Expt 3 because any difference in structure from the ‘intermediate’ treatment would have crested the surface and protruded out of the tank given the dimensions of the reef footprint (45×60 cm). Structural complexity was measured by determining shell volume via water displacement as well as calculating the Sp/Pr index [18]. For the Sp/Pr index, three horizontal and three vertical axes were randomly determined on the reef. The total number of gaps along each axis was counted, and the width or height (depending whether it was on a horizontal or vertical axis), was also measured (to the nearest mm). The gaps were then averaged across both horizontal and vertical axes and divided by the average predator size (operculum to operculum width, to the nearest mm). An Sp/Pr value under 1.0 means the fish predator cannot access the average gap size to reach potential prey within the shell matrix. Each experimental reef covered about 20% of the tank bottom, with the remainder of the tank bare. No structure was used for the control treatments in each experiment (only the predator and prey were in the tank). Pilot runs involving both predator and prey indicated there were no so-called corner effects [15] where prey may be able to hide from predators by aggregating in the corners of the tank.

**Table 1:** Shell volume (L m^−^^2^), prey density (ind. m^−^^2^), and index of ‘predator-free space’ (Sp/Pr) for each experiment by treatment type (n = 5).

	Treatment	Shell volume (L m^−2^)	Prey density (ind. m^−^^2^)	‘Predator-free space’ (Sp/Pr)
**Expt 1**				
	**Control**	0	148148	0
	**Low**	7.4	148	0.61
	**Intermediate**	11.1	148	0.53
	**High**	18.5	148	0.46
**Expt 2**				
	**Control**	0	148	0
	**Low**	7.4	148	0.61
	**Intermediate**	11.1	222	0.53
	**High**	18.5	444	0.46
**Expt 3**				
	**Control**	0	148	0
	**Low**	7.4	148	1.53
	**Intermediate**	11.1	222	1.33

Expt 1 used a fixed number of predator and prey individuals across treatments of variable levels of structural complexity. Expt 2 used the same structural treatments as the first experiment, but scaled the density of prey across treatments. Expt 3 used the same prey density treatments as the second experiment, but altered the amount of ‘predator-free space’ (by increasing interstitial space) in each structural treatment. Control treatments had no structure.

### Experimental Trials

The densities of *P. pugio* used represent a realistic range of densities found in estuarine environments in the northern GOM [36], [45]. Predator density was kept constant (1 individual per tank) for two reasons. One, while the abundance of resident species living within the shell matrix of oyster reefs has been found to increase with reef area or habitat complexity [46], [47], the abundance of transient, predatory species does not always increase [38], [48]. Two, the size of our experimental tanks and reefs would make two red drum difficult to manage, and controlling predator density allowed us to more easily interpret complexity and prey density variations.

All treatments in each experiment were replicated 5 times. Treatments were assigned randomly to experimental tanks and days. Each trial was run for 24 h (beginning at 8 am) and consisted of first partitioning the tank (separating the treatment area) with a barrier. For each trial, randomly selected shrimp were added to the treatment side of the tank and one red drum (starved for 48 h) was added to the empty side. Red drum were randomly selected and used more than once, but never in consecutive trials. We observed no ‘learned’ behaviors in the fish and no individuals were used more than twice. Initial observations indicated that red drum needed time to acclimate to their new surroundings (>1 h), therefore, organisms were allowed 2 h to acclimate before removing the barrier, and the trial allowed interactions for 22 h. After each trial was complete, the red drum was removed followed by the oyster shell. Remaining shrimp were then quantified and removed, and water quality (salinity, temperature, dissolved oxygen, ammonia) measured. Pilot runs with no predator indicated a > 98% recapture rate of shrimp.

### Statistical Analysis

All data were tested for normality and homogeneity of variance; no transformations were necessary. To examine the effects of structure and structural complexity on prey survivorship within each experiment, pair-wise comparisons of least squared means, using a two-way ANOVA, followed by Tukey HSD, were run for each experiment with treatment and day as the factors, and prey survivorship (the proportion of the number of prey initially stocked minus the number of prey recovered) as the response variable. Day was included as a randomized block factor and the model was independent of order. To examine the effects of prey density on prey survivorship, individual contrasts were run between each respective treatment in Expt 1 and 2 (e.g., control Expt 1 vs. control Expt 2; low Expt 1 vs. low Expt 2). To examine the effects of increased interstitial space (or decreased predator-free space) on prey survivorship, individual contrasts were run between Expt 2 and Expt 3 (e.g., low Expt 2 vs. low Expt 3). Data are reported as mean ± 1 SE unless indicated differently.

## Results

**Figure 1:**
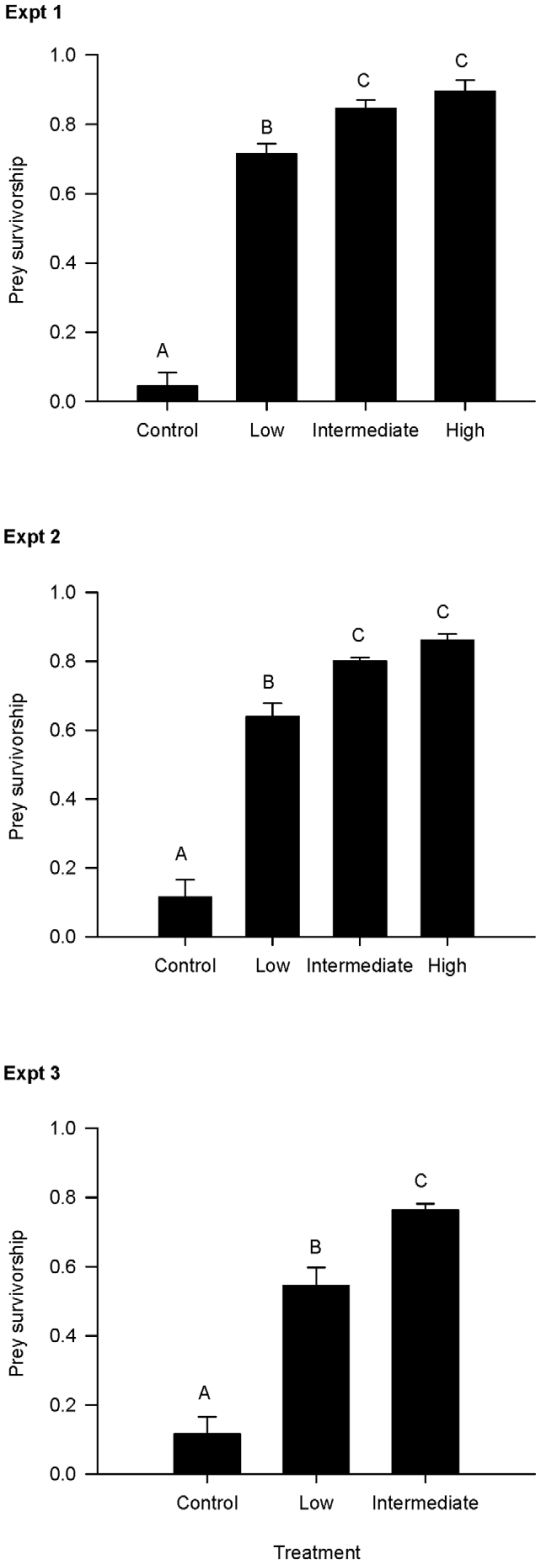
Prey survivorship (no. prey removed/no. prey initially stocked) by experiment. Vertical bars represent prey survivorship (mean ± 1 standard error), or the proportion of prey that survived in each treatment. Different letters represent significant (p<0.05) differences among treatments. Expt 1 used a fixed number of predator and prey individuals across treatments of variable levels of structural complexity. Expt 2 used the same structural treatments as the first experiment, but scaled the density of prey across treatments. Expt 3 used the same prey density treatments as the second experiment, but altered the amount of ‘predator-free space’ (by increasing interstitial space) in each structural treatment. Control treatments had no structure.

In Expt 1, a fixed number of prey (i.e., 40) was added to each treatment and prey survivorship ranged from 4.5 to 89.5% (F_1,16_ = 153.3, p<0.001; Table 2). Prey survivorship was significantly lower in the control (4.5%) treatment than the low treatment (71.5%; p<0.001), and survivorship in both control and low were significantly lower than the intermediate treatment (84.5%; p = 0.01) (Fig. 1). However, prey survivorship did not differ significantly between the intermediate and high treatments (89.5%; p = 0.28). The other factor in the model, day, was not significant.

**Table 2:** Results from each experiment showing the number of prey added (ind), mean number of prey removed (ind ± 1 standard error), mean prey survivorship, and two-way ANOVA results (F-value).

Treatment		Prey added	Prey removed	Prey survivorship	ANOVA result
**Expt 1**					153.3***
	**Control**	40	1.8 (1.6)	0.045	
	**Low**	40	28.6 (1.2)	0.715	
	**Intermediate**	40	33.8 (1.0)	0.845	
	**High**	40	35.8 (1.3)	0.895	
**Expt 2**					103.0***
	**Control**	40	4.6 (2.0)	0.115	
	**Low**	40	25.6 (1.5)	0.640	
	**Intermediate**	60	48.0 (0.6)	0.800	
	**High**	120	103.4 (2.2)	0.861	
**Expt 3**					58.4***
	**Control**	40	4.6 (2.0)	0.115	
	**Low**	40	21.8 (2.1)	0.545	
	**Intermediate**	60	45.8 (2.5)	0.763	

Prey added represents the number of initially stocked individuals per trial. Prey removed represents the number of individuals remaining in the tank after each trial, and the prey survivorship is the number of prey recovered divided by the number of prey added. Expt 1 used a fixed number of predator and prey individuals across treatments of variable levels of structural complexity. Expt 2 used the same structural treatments as the first experiment, but scaled the density of prey across treatments. Expt 3 used the same prey density treatments as the second experiment, but altered the amount of ‘predator-free space’ (by increasing interstitial space) in each structural treatment. Control treatments had no structure. * p<0.01, ** p<0.001, *** p<0.0001.

In Expt 2, the number of prey used was scaled to the same structural treatments as Expt 1 (e.g., increasing structural complexity meant increasing the number of prey individuals used) and prey survivorship ranged from 11.5 to 86.1% (F_1,16_ = 103.0, p<0.001; Table 2). The same trends among treatments in Expt 1 were present in Expt 2: survivorship in the control treatment (11.5%) was significantly lower than the low treatment (64.0%; p<0.001), as well as between the control, and low and intermediate (80.0%) treatments (p<0.01) with no difference between the intermediate and high (86.1%) treatments (p = 0.21) (Fig. 1). The other factor in the model, day, was not significant.

In Expt 3, the number of prey used was scaled as in Expt 2, but low and intermediate structural treatments were changed to increase predator-free space. Prey survivorship ranged from 11.5 to 76.3% (F_1,12_ = 58.4, p<0.001;Table 2) with significant differences between control and low treatments (54.5%; p<0.001), as well as low and intermediate (76.3%; p<0.01) (Fig. 1). The other factor in the model, day, was not significant.

Individual contrasts between treatments in Expt 1 and Expt 2, examining the effects of prey density on survivorship, showed no significant differences at any level of structural complexity (Table 3). Similarly, contrasts between Expt 2 and Expt 3, examining effects of predator-free space showed no significant differences either (Table 3). While not significant (p = 0.055), the contrast between low treatments in Expt 2 and 3 showed a trend of lower survivorship in Expt 3 as compared to Expt 2.

**Table 3:** Results from individual contrasts by experiment (e.g., Expt 1 ‘low’ treatment contrasted with Expt 2 ‘low’ treatment) of prey survivorship between treatments.

Experiment	Contrast	F-value	*p*
1 vs. 2	low	2.43	0.127
1 vs. 2	intermediate	0.87	0.355
1 vs. 2	high	0.48	0.492
2 vs. 3	low	3.90	0.055
2 vs. 3	intermediate	0.58	0.451

Contrasts in Expt 1 and Expt 2 were conducted to examine the effects of prey density on prey survivorship across treatments of increasing structural complexity. Similarly, contrasts between Expt 2 and Expt 3 were conducted across treatments to examine the effects of ‘predator-free space’ on prey survivorship and whether these changed with structural complexity.

## Discussion

Clearly, structure provided by the mesocosm oyster reefs facilitated enhanced prey survivorship. Prey survivorship increased with increasing oyster reef (structural) complexity, but only to a certain point at which point increasing structural complexity failed to further increase survivorship. Neither scaling prey density to structural complexity (shell volume), nor increasing the ‘predator-free space’ (Sp/Pr) significantly changed these findings. This suggests that survivorship of grass shrimp from red drum was dependent on the presence of structure and its complexity (oyster shell volume) more so than effects of predator-free space or density-dependent prey effects. The latter conclusion is based on the assumption that in our experimental mesocosms, increasing the number of prey would increase predator-prey encounter rates when refuge space became limited.

Prey survivorship may be dictated by top-down effects if the ability to detect and escape from predators, or the availability of refuges is a function of habitat structure and its complexity. In estuarine and marine systems, structural complexity associated with vegetation is generally regarded as a feature that increases prey survivorship, presumably through the provision of spatial refuges and by moderating competitive interactions of predators [49], [50]. However, complex habitats may not always benefit prey, especially if a prey's ability to escape is impeded by the structure [13] or density-dependent effects related to encounter rates are present [22]. Similar patterns exist in terrestrial systems where certain prey might choose accessibility to escape routes over potential concealment; for example, woody vegetation has been shown to have both positive and negative effects on predation risk [51], [52]. The physical structure provided by the oyster reefs in this study differs markedly from other structural features which may be flexible (i.e. seagrass) and represent only a visual barrier to predator detection. The solid nature of the oyster shell reefs may impede not only predator vision, but also movement and access. It may therefore be the functional characteristics of physical structure (e.g., flexible, malleable, or solid substrate) which are most important in dictating prey survivorship and therefore make comparisons across studies difficult.

The influence of habitat structure on prey survivorship within a community is complex. Our results generally agree with theory that habitat structure and its complexity increase prey survivorship to a point [20]. However, because of the scale and artificial nature of laboratory experiments, generalization of these findings should be done with caution. In a natural environment, while the presence of oyster reefs may be able to support a greater biomass of associated nekton species compared to unstructured habitat, it may not always mean that increasing complexity provides superior habitat [53]. For instance, multiple-predator effects can often have important and very different consequences for their prey through interference interactions as well as intra- and interspecific competition, and may in fact override structural differences within habitats [54], [55]. A series of studies examining multiple predator effects in simple and complex created oyster reefs found that the effects of complexity may vary depending on predator identity when more than one predator is considered [6], [55], [56]. In this study, we conducted a mesocosm experiment with a simplified food web (using only one predator and one prey species). Our findings may differ given a different predator-prey combination, or by adding other predators.

Scaling predator and/or prey abundance with habitat complexity may better mimic conditions found in nature [22]. Recent studies showed that vegetated treatments which simultaneously increased complexity and predator and prey densities, did not consistently result in differences in prey survivorship [23]. One possible explanation for this is that complexity might have decreased foraging efficiency, but whatever increase in survivorship resulting from increased complexity was cancelled out by having more predators and prey. As a result, this may have increased prey encounter rates. However, encounter rates can only be inferred as direct observations of behavior are absent. While these experiments may mimic nature better than others by confounding influences, conclusions from these experiments are difficult and some might consider them impossible. When we scaled only prey density with complexity, we found no significant difference in prey survivorship when compared to not increasing prey density. This suggests that prey survivorship in our experiment may not be dependent on prey density, and that the amount of refuge space may be the limiting factor. As stated previously, this is assuming that encounter rates would increase when refuge space became limiting. However, another possibility is that we may have failed to reach any potential threshold for prey density.

For oyster reefs, numerous factors can contribute to the complexity of the habitat; along with shell volume, interstitial space has been cited as a primary factor providing valuable prey refuge habitat [47]. This space in-between structure is hypothesized to provide refuge for prey as long as the size of the gap in the structure is large enough for the prey to fit, but small enough so that the predator cannot [31]. Bartholomew et al. [18] hypothesized a “threshold” response in prey survivorship to decreasing Sp/Pr. At very high Sp/Pr, prey survivorship should be uniformly low. At very low Sp/Pr, prey survivorship should be uniformly high (as long as prey can fit through the spaces in the habitat) with a rapid transition between the two states. In theory, Sp/Pr greater than 1 indicates that the predator can access all space in the structure, while Sp/Pr less than 1 indicates refugia for prey. However, it is not clear at what point predators may actually access space, and it is likely that the actual value at which predators are excluded from space may not be 1. One methodological consideration is that a more accurate indicator of refuge space might require ‘predator-free space’ measures that also account for the available area within the structure itself (i.e., volume) and not just on the surface of a structure. This would tell us more about the nature of the refuge space available to prey, especially for 3-dimensional, heterogeneous structures like oyster reefs.

While we compared Sp/Pr values greater than 1 (1.53, 1.33) to values less than 1 (0.61, 0.53), we failed to detect significant decreases in prey survivorship, although there was a trend of lower survivorship with Sp/Pr > 1. It is possible that the differences tested failed to compare a large enough gradient in ‘predator-free space’ to detect significant effects; the oyster shell reefs we created had Sp/Pr values just over 1 and we may have not reached the “threshold” in ‘predator-free space’. Again, it appears that the type of habitat used to create structure, and potentially predator and prey identity, may be critical to the outcome. For example, seagrass provides complexity in the habitat but with movable parts, and this obviously has different implications for predator access than habitat composed of solid structure (e.g., oyster shell). Furthermore, it is possible that regardless of the size of the predator, their ability and likelihood of entering tight spaces may be low and vary greatly by species.

The results of this study add to our understanding of the role of habitat structure in mediating prey survivorship. Specifically, the results corroborate theory that prey survivorship can increase as structure is introduced and made more complex, but with diminishing returns [18]. This general pattern of prey survivorship was not significantly affected by changes in prey density or amount of ‘predator-free space’. Future studies should aim to use functionally different substrate types (e.g., substrate with different physical properties) and combinations of predators and prey to more accurately describe patterns across systems and better mimic nature. Also, new designs should aim to create larger gradients in ‘predator-free space’ that incorporate within-structure metrics and be on more ecologically relevant scales to bridge theory with reality.
